# Integration of Public Health in LCME Accredited Medical Schools in Florida: A Survey Based Study

**DOI:** 10.7759/cureus.5213

**Published:** 2019-07-23

**Authors:** Joan E St. Onge, Ann M Cata, Viviana E Horigian

**Affiliations:** 1 Internal Medicine, Graduate Medical Education, University of Miami Miller School of Medicine, Miami, USA; 2 Miscellaneous, Graduate Medical Education, University of Miami Miller School of Medicine, Miami, USA; 3 Public Health Sciences, University of Miami Miller School of Medicine, Miami, USA

**Keywords:** medical curriculum, public health, competencies, teaching methods, community health

## Abstract

Introduction: The future physician will face a career challenged by a number of significant changes in healthcare, including changes in demographics and disease, an increasing focus on population health and value-based care, and changes in healthcare funding. National organizations have called for medical schools to better prepare students for these challenges, and to incorporate more public health education in medical school. While many medical schools have responded, the topics covered, the timing in the curriculum, and the importance of these topics for graduation vary widely. Florida has been a site of growth in medical education in the last 10 years. Given that new medical schools were developed during a period of increased emphasis on the need for public health education, a survey was developed to assess the state of public health education in medical schools accredited by the Liaison Committee for Medical Education (LCME) in the state of Florida.

Methods: The survey included questions on school location, size of the student body, date of initial LCME accreditation, presence of department or school of public health, and presence of a pathway or track in public health. The survey asked detailed questions about public health content, curricula delivery methods, and timing of the curriculum within the four-year course of study as well as the courses providing public health content. The survey asked about the value of curricular content and the survey itself. The online survey was sent to the associate or senior associate deans for education in the seven LCME accredited schools who had full or provisional accreditation as of December 2017. Data collection occurred between March 14 and March 30, 2018.

Results: Six of the seven medical schools responded. Of the eleven competencies included in the survey, schools reported between five and eleven. Three schools cover nine or more of the eleven competencies. The number of competencies covered was not statistically influenced by age of the school, percentage of underrepresented students in medicine, the presence of a school or department of public health, or a special pathway or track in public health. The most common teaching method used was a didactic lecture, and the least was the structured experience with a local health organization. The fourth year of medical school saw the least amount of public health education. Five of the six respondents felt that the competencies presented here are very important to extremely important, and one school feels that they are moderately important.

Discussion: Nationally, education in public health is an important component in medical education, but the topics included, educational methods used and the importance of the content varies from school to school. The state of public health education in medical schools in Florida is robust in some schools. The individuals responsible for the curriculum support the importance of these topics. The content is delivered through a diversity of pedagogical methods. The study results demonstrate a number of opportunities for enhancement.

Conclusion: Given the importance of public health content in medical schools, survey methodology using established competencies to assess public health curricula could be used in the US to provide an up-to-date assessment of the strengths and opportunities for improvement in this area.

## Introduction

The future physician faces a career challenged by changing demographics and disease patterns, a responsibility to provide high-quality healthcare at low cost, an ever-changing payment and insurance landscape, and an enlarging gap in the quality of health among different populations. There is a growing appreciation for the model of social determinants’ of health influence on the wellbeing of individuals and populations. A 1999 Institute of Medicine (IOM, now the National Academy of Medicine) report proposed a model of determinants that illustrated how individual characteristics and environmental characteristics influence health-related quality of life such as symptoms, functional status, health perceptions, and opportunity. In this model, individual characteristics are biology, life course, lifestyle and health behavior, illness behavior, and personality and motivation; environmental characteristics include the social and cultural influences of health [1]. Marmot, in an article announcing the World Health Organization’s Commission on Social Determinants of Health, lists 10 important social determinants of health inequalities, including stress, social exclusion, social support, work and unemployment, addiction and transportation among others [2]. The 2003 IOM report, “Who Will Keep the Public Healthy?” calls for the incorporation of public health topics in the medical school curriculum. To improve health, the future will require physicians who are skilled team players and excel in systems-based practice, who provide patient-centered care and can work with and in their communities [3]. The commission advocates that efforts should be undertaken by academic health centers to provide joint classes and clinical training in public health and medicine. Finally, the report calls for “a significant proportion of medical school graduates should be fully trained in the ecological approach to public health at the Master in Public Health level" [3]. As of June 2010, the Liaison Committee for Medical Education (LCME), the body that sets accreditation standards for MD-granting medical schools, explicitly includes education in public health sciences and preventive medicine [4]. In a recent position paper, the American College of Physicians recommends, “social determinants of health and the underlying individual, community, and systemic issues related to health inequities be integrated into medical education at all levels" [5]. In graduate medical education, the Accreditation Council for Graduate Medical Education (ACGME), the accrediting body for residency programs in the United States, calls for the incorporation of interprofessional quality improvement activities that focus on reducing healthcare disparities [6].

Medical education that focuses solely on experience in individual patient care is not sufficient to prepare the future physician to practice high-quality, cost-effective care. In a 2010 review of critiques of medical practice in the United States pertaining to quality, evidence-based medicine, population medicine, health policy and heuristics, Sales and Schlaff found that “physicians are inadequately trained to function in the complex organizational and social systems that characterize modern practice" [7]. Academic health centers have the experience, facilities, and research capabilities to guide this transformation from traditional delivery models to those that will focus on value, safety, and the health of populations [8]. Medical education is important and must continue to be an integral part of this transformation.

National collaborations have taken place to improve public health curricula in medical schools. A 2003 partnership between the Association of American Medical Colleges (AAMC) and the Centers for Disease Control (CDC) resulted in the formation of The Regional Medicine-Public Health Education Centers (RMPHEC) initiative, aimed at improving education in population health, public health, and prevention for medical students and residents [9]. These schools were required to collaborate with state and local public health agencies in this effort. In 2013, the American Medical Association (AMA) launched the Accelerating Change in Medical Education Initiative (ACE) aimed at training physicians “to meet the needs of today's patients and to anticipate future changes" [10]. The program now has 32 medical school partners.

The United States is not the only country addressing the need for more public health education in medical schools. In 1996, the academic departments of public health in the United Kingdom held a meeting to address how to increase the interest of clinical students in public health. The group suggested a number of topics to engage students, including international public health, health policy, environmental health, and the use of data and analysis to impact public health [11]. Canadian medical schools have been incorporating public health curricula in medical education since 2001, but subsequent student focus groups demonstrated a lack of satisfaction with the curriculum [12].

US Medical School Public Health Curricula

A literature review indicates that medical schools in the United States have public health topics in their curricula. However, the topics presented, the number of contact hours, the importance of this content for graduation, and the timing across the four years of study differ from school to school. In addition to biostatistics and epidemiology, topics include healthcare disparities, servant leadership, cultural competency, advocacy, population health, quality and patient safety, community-based research, environmental health, and health policy. Schools offer this curriculum through elective experiences, orientation requirements, as part of a required course in the preclinical curriculum, or as part of a third-year or fourth-year clinical rotation. Some offer longitudinal experiences in all four years for selected students, while a few require this type of experience for all students. A number of schools have separate tracks for a predetermined group of students on regional campuses. (JES, unpublished observations, 2017)

The results of the courses have been positive. Course outcomes demonstrate an increase in factual knowledge, and that content is of high value to students [8,13-14]. A number of schools target specific patient populations through integrating lectures and cases into the ongoing curriculum with positive results [15-16]. Delivery of the content occurs using a number of pedagogical approaches. Didactic lectures followed by small group case-based sessions is a commonly used method. Case discussions are used to address public health issues in local communities. Longitudinal tracts used experiential learning with the aforementioned methods. Some schools used a hybrid of these methods [8,15-17]. Results from the LCME and AAMC’s graduation questionnaire indicate that more schools are including a broader range of public health topics in their curricula and that students are increasingly more satisfıed with the public health education that they are receiving [4]. Questions remain regarding appropriate curricular content, the timing of delivery and assessment of the content, as well as the perceived importance of this content to the medical schools.

The Florida experience

Florida has been a site of tremendous growth in medical education over the last 10 years, spurred by a number of facts. In 2008, Florida had the eighth largest percentage of physicians who are of age 60 or older and ranked 36th in the United States in medical school enrollment per 100,000 population [18]. The population in the state of Florida continues to grow; now being the third most populous state in the United States [19]. Expansion of medical school classes and the addition of regional campuses has occurred at some schools. Three new medical schools opened between 2010 and 2017 [20]. While all of the schools have LCME accreditation, the curricular delivery and training opportunities vary, reflecting a variety of pedagogical methods and differing missions of the medical schools. With these rapid developments occurring during a period of increased emphasis on public health education in medical school, Florida is a perfect microcosm to study the state of public health curricular development.

The primary goal of this project is to provide data on the current state of public health education for medical students enrolled in LCME schools in the state of Florida. This information will help set the course for the future development of public health curricula and offer opportunities for collaboration among these schools. The information will provide an overview for medical educators and policymakers regarding the state of public health education in medical schools in Florida. Feedback from the respondents will inform a larger study of US schools planned for the future by the authors.

## Materials and methods

This cross-sectional study uses a survey distributed between March 14, 2018, and April 5, 2018, to medical schools in Florida via the Qualtrics survey platform. The survey includes Florida LCME accredited schools with full or provisional accreditation as of December 13, 2017 [20]. Schools with preliminary accreditation or applications pending were not included in the survey group. Included in the questions are school location, size of student's body, date of initial LCME accreditation, presence of a department or a school of public health, and presence of a pathway or a track in public health. In addition, questions about public health content, curricula delivery methods, the timing of the curriculum in the four-year course of study, and the titles of courses providing public health content. Respondents were asked to include information about the medical curriculum only, not that which is presented as requirements for a Master in Public Health degree (see Appendix A).

Survey questions regarding public health education reflect the competencies found in Maeshiro’s article, “Medical Education for a Healthier Population: Reflections on the Flexner Report from a Public Health Perspective" [21]. These competencies for medical students were used in the Regional Medicine-Public Health Initiative sponsored by the AAMC and the Centers for Disease Control [9]. Survey distribution took place via email. The initial distribution included an introductory email and a follow-up email reminder one week later. This study is exempted from Institutional Review Board approval, based upon the federal definition of research pursuant to 45 CFR 46, and the “Not human subject self-certification research tool” of the University of Miami Human Subjects Research Office.

Statistical analysis

The age of the school, the presence of a school or department of public health, the presence of a pathway in public health, the percentage of students underrepresented in medicine, and the importance of the public health curriculum were compared with the number of competencies covered by the school’s curriculum. This was analyzed via a one-tailed t-test for equality of means. Timing of educational opportunities in the curriculum is presented in an aggregate form. School’s pedagogical methods are reported as a percent of the total number of encounters available for each competency as calculated by 

*Actual number of teaching methods used to teach individual competencies* divided by (*the total teaching opportunities or seven teaching methods *multiplied by *the number of schools teaching each competency*).

The survey included questions on the importance of the public health competencies, the usefulness of the collected data, and about the survey itself.

## Results

Of the seven eligible medical schools, six completed the survey, with an 85% response rate. The newest medical school was accredited in 2015, and the oldest in 1955. The number of students enrolled at each school varies between less than 100 to greater than 751. Four schools have student bodies of 451-600. The percentage of underrepresented students at each school varies from 6%-10% to greater than 30%. None of the schools is in a rural area; five describe their location as suburban and two as urban. Four schools have a school or department of public health, and three of the schools offer a special track or pathway in public health, with up to 10% of the students participating in these tracks. 

The number of competencies in the curriculum at each school ranges from five to eleven of the eleven competencies listed in the survey. Three of the schools report offering educational opportunities to address nine or more competencies. There is no statistical difference in the number of competencies in the curriculum compared with the presence of a department of public health or a pathway in public health. Schools with a department or school of public health address an average of nine competencies (SD = 2.71); schools without this address an average of seven of the eleven competencies (SD = 1.41). Schools offering a special track or pathway in public health do not differ from those who do not offer this track. Each offer educational opportunities at an average of 8.33 competencies. There is no difference in the number of competencies addressed in the curriculum based upon years since accreditation or percentage of enrolled students underrepresented in medicine (Table 1).

**Table 1:** Characteristics of Florida medical schools and the number of competencies addressed

	Yes Mean, SD	No Mean, SD	
Department or school of public health	9.0, 2.71	7.0, 1.41	p = 0.2
Pathway or track in public health	8.33, 2.88	8.33 2.51	p = 0.5
Date of initial LCME accreditation before 2010	8 (4.24)	8 (2)	p = 0.5
Percent of enrolled students who are underrepresented in medicine is greater than 15%	8.0 (3.0)	8.66 (2.31)	p = 0.39

 

All schools offer educational opportunities related to the following competencies: appraise the quality of the evidence of peer reviewed medical and public health literature, apply primary and secondary prevention status that improves the health of individuals and populations; and describe the organization and financing of the US healthcare system and their effects on access, utilization, and quality of care. Educational opportunities to address the competency, "participate in population health improvement strategies," is present in a few schools (Table 2).

**Table 2:** Number of Florida medical schools offering educational opportunities for the listed public health competency

Competency	n (%)
Assess the health status of populations using available data	4 (67%)
Integrate emerging information on individuals' biologic and genetic risk with the population	4 (67%
Appraise the quality of the evidence of peer-reviewed medical and public health literature	6 (100%)
Apply primary and secondary prevention status that improve the health of individuals and populations	6 (100%)
Identify community assets and resources to improve the health of individuals and populations	4 (67%)
Explain how community engagement strategies improve health and contribute to the reduction of health disparities	4 (67%)
Participate in population health improvement strategies	3 (50%)
Discuss the function of public health systems	5 (83%)
Describe the organization and financing of the US healthcare system re-access, utilization, and quality of care	6 (100%)
Discuss the ethical implications of health care resource allocation and emerging technologies on population health	5 (83%)
Identify quality improvement methods to improve medical care and population health	4 (67%)

**Figure 1:**
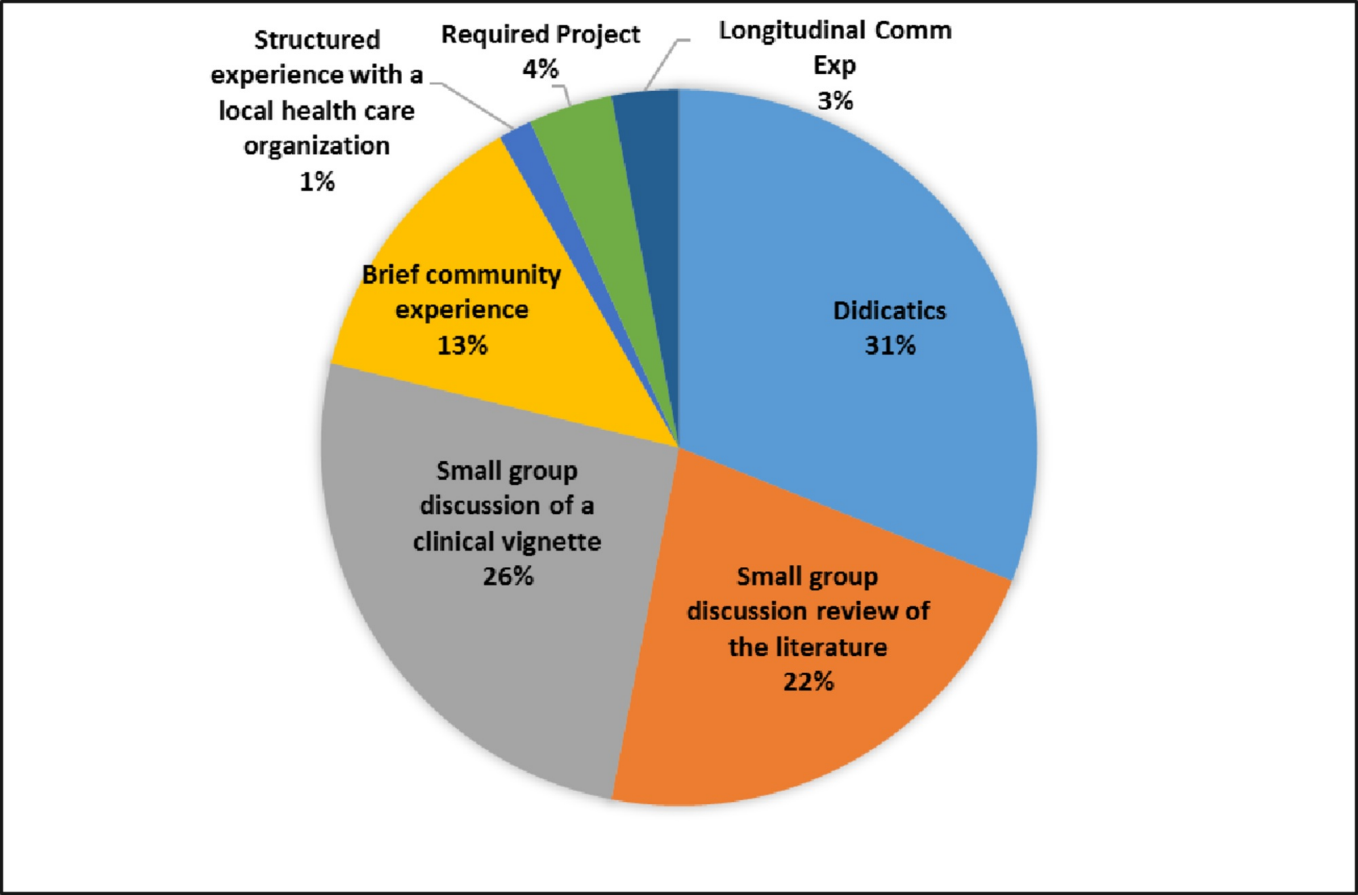
Teaching methods utilized to teach public health competencies in Florida medical schools

**Figure 2:**
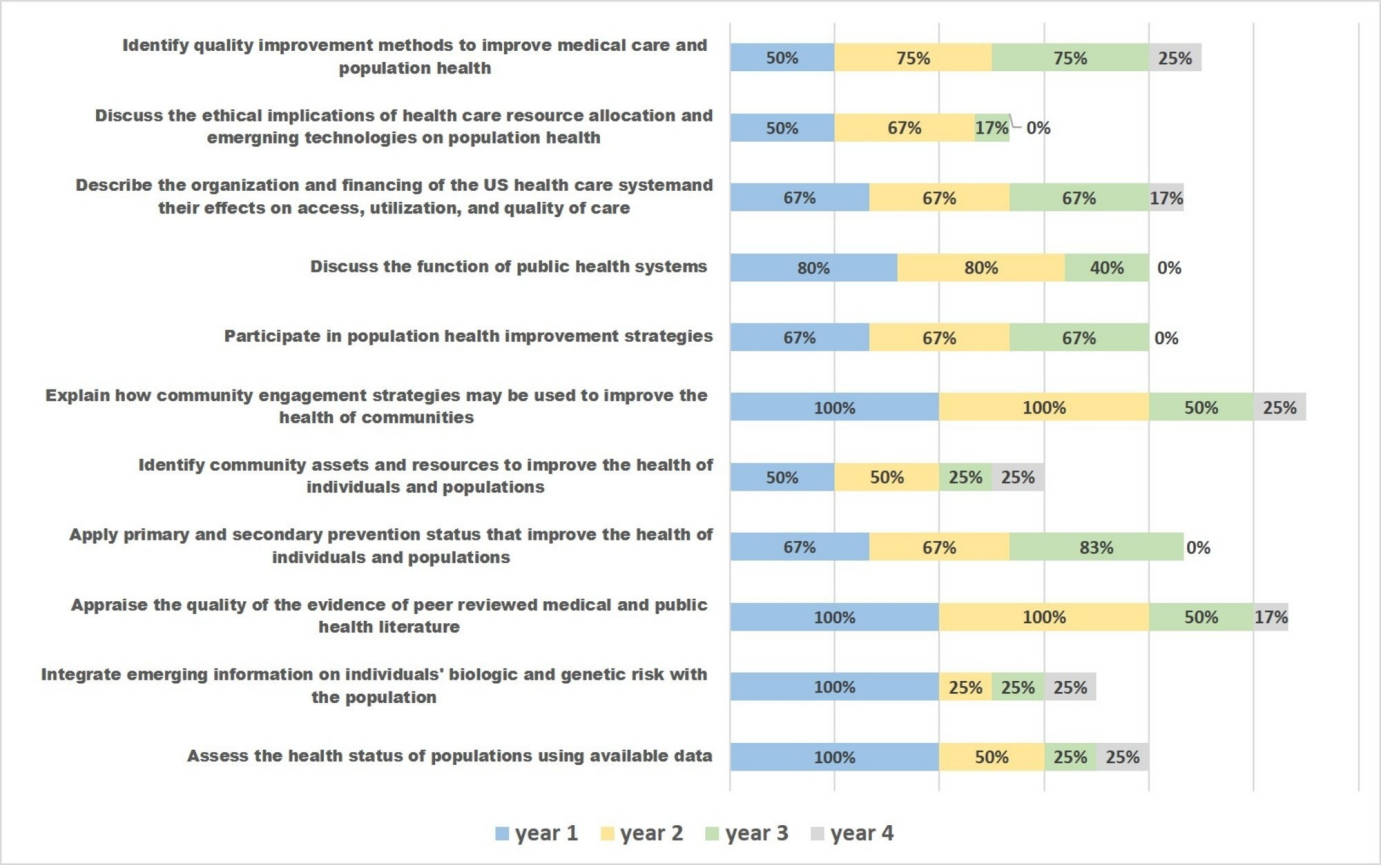
Percentage of schools presenting each compentency by year in curriculum

The educational methods employed to teach the competencies varies. The most common method is the small group discussion, either focused on a clinical vignette or review of the literature, followed by the use of didactic lectures. The structured experience with a local healthcare organization option is the least frequently used method (Figure 1). Timing of the delivery of educational content related to public health varies from school to school. As seen in Figure 2, the percentage of curriculum taught in Year 1, Year 2, Year 3, and Year 4 varied by competency. For instance, the competency of "identify quality improvement methods to improve medical care and population health" is presented in Year 1 by 50%, Year 2 in 75%, Year 3 by 75% and in Year 4 by 25% of the schools who address this competency. This is compared to the competency "discuss the function of the public health system" which is presented in Year 1 by 80%, Year 2 by 80% and in Year 3 by 40% and in year 4 by none of the schools who address this competency. Five of the six respondents feel the competencies presented here are “very important” to “extremely important,” and one school feels that they are “moderately important.” Respondents recommend addition of a longitudinal option for the timing in the curriculum, and allowing the schools to list more than one location for clinical experiences.

## Discussion

The state of public health education in Florida medical schools is robust in some schools. A diversity of pedagogical methods is used to deliver this content. There are educational opportunities focusing on many of the public health competencies. While the schools vary in the number of competencies in the curricula, the majority of the schools offer education related to 70% or more of the public health competencies studied here. The age of the school, the presence of a department of public health or pathway in public health, and the percentage of underrepresented students in medicine do not affect the degree to which these competencies are present in a school’s curriculum. 

While there is diversity in the pedagogical methods in place, a commonly used method, the didactic lecture, is the most passive. Schools underutilize learning opportunities in the community. The majority of the public health content is in the first three years of medical school. It is unclear why the fourth year, with time for electives and for other pursuits, is not utilized more. This is an ideal year to incorporate this content and may offer an opportunity for more active learning to reinforce the public health content offered earlier in the curriculum. Projects and structured experiences with community organizations are options to teach these competencies, especially in the fourth year, as the student looks forward to residency training and more self-directed learning. In addition, longitudinal threads or themes in the curriculum are ways to incorporate public health in all four years of medical education. 

The strengths of this study are the response rate and the focus on established competencies of public health. The respondents are actively engaged in the curriculum of their medical schools and their responses should reflect the true status of public health education at their school. While the study sample is small, it adequately reflects the current state of public health education in LCME accredited medical schools in Florida. The limitations include the lack of generalizability of the findings beyond Florida and the use of a non-validated survey instrument. 

## Conclusions

Public health education in medical schools in Florida reflects the growing importance of this content for physicians. Newer and older schools have been equally engaged in offering public health topics in their curriculum. There are opportunities to enhance this content and the methods of teaching to ensure that all students are knowledgeable about the tenets of public health, so they may be and are prepared for this changing profession. This study is a model that can be used to ascertain the status of public health education in all medical schools across the United States.

## Appendices

Appendix A

Please note:  *Skip Logic is presented in italics.* 

Q1 How many total students are enrolled in your school? 

o 100  (1)

o 101-240  (2)

o 241-300  (3)

o 301-450  (4)

o 451-600  (5)

o 601-750  (6)

o 751 or greater  (7)

Q2 When was your school accredited?

________________________________________________________________

Q3 How would you describe the location of your medical school?

o Urban  (1)

o Suburban  (2)

o Rural  (3)

Q4 How would you describe the location where the clinical clerkships occur?

o Urban  (1)

o Suburban  (2)

o Rural  (3)

Q5 Of the total number of students currently enrolled at your school, what is the percentage of students who are underrepresented in medicine?

o 0-5%  (1)

o 6-10%  (2)

o 11-15%  (3)

o 16-20%  (4)

o 21-25%  (5)

o 26-30%  (6)

o Greater than 30%  (7)

Q6 Does your university or medical school include a school or department of public health?

o Yes  (30)

o No  (31)

Q7 Does your school offer a special track or pathway in public health?  Please note: This does not include students in dual degree programs (MD/MPH or MD/MPSH programs). 

o Yes  (1)

o No  (2)

Display This Question:

If Does your school offer a special track or pathway in public health?  Please note: This does not i... = Yes

Q8 Please describe the pathway or track. 

________________________________________________________________

Display This Question:

Does your school offer a special track or pathway in public health?  Please note: This does not i... = Yes

Q9 What percentage of your students participate in the track?

o 0-5%  (1)

o 6-10  (2)

o 11-15%  (3)

o 16-20%  (4)

o 21-25%  (5)

o greater than 25%  (6)

Q10 Does your medical school curriculum offer opportunities to address the following public health competency:  Assess the health status of populations using available data (e.g., public health surveillance data, vital statistics, registries, surveys, electronic health records and health plan claims data)?

o Yes  (1)

o No  (2)

Display This Question:

If Does your medical school curriculum offer opportunities to address the following public health co... = Yes

Q11 During what year is the content offered? ( choose all that apply)

▢ Year 1  (1)

▢ Year 2  (2)

▢ Year 3  (3)

▢ Year 4  (4)

Display This Question:

If Does your medical school curriculum offer opportunities to address the following public health co... = Yes

Q12 Please list the course or courses that address this competency. 

________________________________________________________________

Display This Question:

If Does your medical school curriculum offer opportunities to address the following public health co... = Yes

Q13 Please indicate the teaching methods used to deliver this content:

▢ Didactic Lecture  (1)

▢ Small group discussion focusing on review of the literature  (2)

▢ Small group discussion focusing on a clinical vignette  (3)

▢ Brief experiential learning opportunity in the community  (4)

▢ Structured experience with a local health system  (5)

▢ Required project  (6)

▢ Longitudinal experience in the community  (7)

Q14 Does your medical school curriculum offer opportunities to address the following public health competency:  Integrate emerging information on individuals’ biologic and genetic risk with population level factors when deciding upon prevention and treatment options.

o Yes  (1)

o No  (2)

Display This Question:

If Does your medical school curriculum offer opportunities to address the following public health co... = Yes

Q15 During what year is the content offered? ( choose all that apply)

▢ Year 1  (1)

▢ Year 2  (2)

▢ Year 3  (3)

▢ Year 4  (4)

Display This Question:

If Does your medical school curriculum offer opportunities to address the following public health co... = Yes

Q16 Please list the course or courses that address this competency. 

________________________________________________________________

Display This Question:

If Does your medical school curriculum offer opportunities to address the following public health co... = Yes

Q17 Please indicate the teaching methods used to deliver this content:

▢ Didactic Lecture  (1)

▢ Small group discussion focusing on review of the literature  (2)

▢ Small group discussion focusing on a clinical vignette  (3)

▢ Brief experiential learning opportunity in the community  (4)

▢ Structured experience with a local health system  (5)

▢ Required project  (6)

▢ Longitudinal experience in the community  (7)

Q18 Does your medical school curriculum offer opportunities to address the following public health competency:  Appraise the quality of the evidence of peer reviewed medical and public health literature   and its implications at patient- and population- levels?

o Yes  (1)

o No  (2)

Display This Question:

If Does your medical school curriculum offer opportunities to address the following public health co... = Yes

Q19 During what year is the content offered? ( choose all that apply)

▢ Year 1  (1)

▢ Year 2  (2)

▢ Year 3  (3)

▢ Year 4  (4)

Display This Question:

If Does your medical school curriculum offer opportunities to address the following public health co... = Yes

Q20 Please list the course or courses that address this competency. 

________________________________________________________________

Display This Question:

If Does your medical school curriculum offer opportunities to address the following public health co... = Yes

Q21 Please indicate the teaching methods used to deliver this content:

▢ Didactic Lecture  (1)

▢ Small group discussion focusing on review of the literature  (2)

▢ Small group discussion focusing on a clinical vignette  (3)

▢ Brief experiential learning opportunity in the community  (4)

▢ Structured experience with a local health system  (5)

▢ Required project  (6)

▢ Longitudinal experience in the community  (7)

Q22 Does your medical school curriculum offer opportunities to address the following public health competency:  Apply primary and secondary prevention strategies that improve the health of individuals  and populations?

o Yes  (1)

o No  (2)

Display This Question:

If Does your medical school curriculum offer opportunities to address the following public health co... = Yes

Q23 During what year is the content offered? ( choose all that apply)

▢ Year 1  (1)

▢ Year 2  (2)

▢ Year 3  (3)

▢ Year 4  (4) 

Display This Question:

If Does your medical school curriculum offer opportunities to address the following public health co... = Yes

Q24 Please list the course or courses that address this competency.

________________________________________________________________

Display This Question:

If Does your medical school curriculum offer opportunities to address the following public health co... = Yes

Q25 Please indicate the teaching methods used to deliver this content:

▢ Didactic Lecture  (1)

▢ Small group discussion focusing on review of the literature  (2)

▢ Small group discussion focusing on a clinical vignette  (3)

▢ Brief experiential learning opportunity in the community  (4)

▢ Structured experience with a local health system  (5)

▢ Required project  (6)

▢ Longitudinal experience in the community  (7)

Q26 Does your medical school curriculum offer opportunities to address the following public health competency:  Identify community assets and resources to improve the health of individuals and  populations?

o Yes  (1)

o No  (2)

Display This Question:

If Does your medical school curriculum offer opportunities to address the following public health co... = Yes

Q27 During what year is the content offered? ( choose all that apply)

▢ Year 1  (1)

▢ Year 2  (2)

▢ Year 3  (3)

▢ Year 4  (4)

Display This Question:

If Does your medical school curriculum offer opportunities to address the following public health co... = Yes

Q28 Please list the course or courses that address this competency.

________________________________________________________________

Display This Question:

If Does your medical school curriculum offer opportunities to address the following public health co... = Yes

Q29 Please indicate the teaching methods used to deliver this content:

▢ Didactic Lecture  (1)

▢ Small group discussion focusing on review of the literature  (2)

▢ Small group discussion focusing on a clinical vignette  (3)

▢ Brief experiential learning opportunity in the community  (4)

▢ Structured experience with a local health system  (5)

▢ Required project  (6)

▢ Longitudinal experience in the community  (7)

Q30 Does your medical school curriculum offer opportunities to address the following public health competency:  Explain how community-engagement strategies may be used to improve the health of  communities and to contribute to the reduction of health disparities?

o Yes  (1)

o No  (2)

Display This Question:

If Does your medical school curriculum offer opportunities to address the following public health co... = Yes

Q31 During what year is the content offered? ( choose all that apply)

▢ Year 1  (1)

▢ Year 2  (2)

▢ Year 3  (3)

▢ Year 4  (4)

Display This Question:

If Does your medical school curriculum offer opportunities to address the following public health co... = Yes

Q32 Please list the course or courses that address this competency.

________________________________________________________________

Display This Question:

If Does your medical school curriculum offer opportunities to address the following public health co... = Yes

Q33 Please indicate the teaching methods used to deliver this content:

▢ Didactic Lecture  (1)

▢ Small group discussion focusing on review of the literature  (2)

▢ Small group discussion focusing on a clinical vignette  (3)

▢ Brief experiential learning opportunity in the community  (4)

▢ Structured experience with a local health system  (5)

▢ Required project  (6)

▢ Longitudinal experience in the community  (7)

Q34 Does your medical school curriculum offer opportunities to address the following public health competency:  Participate in population health improvement strategies (e.g., systems and policy  advocacy, program or policy development, or other community-based interventions).

o Yes  (1)

o No  (2)

Display This Question:

If Does your medical school curriculum offer opportunities to address the following public health co... = Yes

Q35 During what year is the content offered? (Choose all that apply.)

▢ Year 1  (1)

▢ Year 2  (2)

▢ Year 3  (3)

▢ Year 4  (4)

Display This Question:

If Does your medical school curriculum offer opportunities to address the following public health co... = Yes

Q36 Please list the course or courses which address this competency. 

________________________________________________________________

Display This Question:

If Does your medical school curriculum offer opportunities to address the following public health co... = Yes

Q37 Please indicate the teaching methods used to deliver this content.  (Choose all that apply.)

▢ Didactic Lecture  (1)

▢ Small group discussion focusing on review of the literature  (2)

▢ Small group discussion focusing on a clinical vignette  (3)

▢ Brief experiential learning opportunity in the community  (4)

▢ Structured experience with a local health system  (5)

▢ Required project  (6)

▢ Longitudinal experience in the community  (7)

Q38 Does your medical school curriculum offer opportunities to address the following public health competency:  Discuss the functions of public health systems including those that require or benefit  from the contribution of clinicians, such as public health surveillance, preparedness, and  prevention of chronic conditions?

o Yes  (1)

o No  (2)

Display This Question:

If Does your medical school curriculum offer opportunities to address the following public health co... = Yes

Q39 During what year is the content offered? (Choose all that apply.)

▢ Year 1  (1)

▢ Year 2  (2)

▢ Year 3  (3)

▢ Year 4  (4)

Display This Question:

If Does your medical school curriculum offer opportunities to address the following public health co... = Yes

Q40 Please list the course or courses which address this competency. 

________________________________________________________________

Display This Question:

If Does your medical school curriculum offer opportunities to address the following public health co... = Yes

Q41 Please indicate the teaching methods used to deliver this content.  (Choose all that apply.)

▢ Didactic Lecture  (1)

▢ Small group discussion focusing on review of the literature  (2)

▢ Small group discussion focusing on a clinical vignette  (3)

▢ Brief experiential learning opportunity in the community  (4)

▢ Structured experience with a local health system  (5)

▢ Required project  (6)

▢ Longitudinal experience in the community  (7)

Q42 Does your medical school curriculum offer opportunities to address the following public health competency:  Describe the organization and financing of the U.S. health care system, and their effects  on access, utilization, and quality of care for individuals and populations?

o Yes  (1)

o No  (2)

Display This Question:

If Does your medical school curriculum offer opportunities to address the following public health co... = Yes

Q43 During what year is the content offered? (Choose all that apply.)

▢ Year 1  (1)

▢ Year 2  (2)

▢ Year 3  (3)

▢ Year 4  (4)

Display This Question:

If Does your medical school curriculum offer opportunities to address the following public health co... = Yes

Q44 Please list the course or courses which address this competency. 

________________________________________________________________

Display This Question:

If Does your medical school curriculum offer opportunities to address the following public health co... = Yes

Q45 Please indicate the teaching methods used to deliver this content.  (Choose all that apply.)

▢ Didactic Lecture  (1)

▢ Small group discussion focusing on review of the literature  (2)

▢ Small group discussion focusing on a clinical vignette  (3)

▢ Brief experiential learning opportunity in the community  (4)

▢ Structured experience with a local health system  (5)

▢ Required project  (6)

▢ Longitudinal experience in the community  (7)

Q46 Does your medical school curriculum offer opportunities to address the following public health competency:  discuss the ethical implications of health care resource allocation and emerging  technologies on population health?

o Yes  (1)

o No  (2)

Display This Question:

If Does your medical school curriculum offer opportunities to address the following public health co... = Yes

Q47 During what year is the content offered? (Choose all that apply.)

▢ Year 1  (1)

▢ Year 2  (2)

▢ Year 3  (3)

▢ Year 4  (4)

Display This Question:

If Does your medical school curriculum offer opportunities to address the following public health co... = Yes

Q48 Please list the course or courses which address this competency. 

________________________________________________________________

Display This Question:

If Does your medical school curriculum offer opportunities to address the following public health co... = Yes

Q49 Please indicate the teaching methods used to deliver this content.  (Choose all that apply.)

▢ Didactic Lecture  (1)

▢ Small group discussion focusing on review of the literature  (2)

▢ Small group discussion focusing on a clinical vignette  (3)

▢ Brief experiential learning opportunity in the community  (4)

▢ Structured experience with a local health system  (5)

▢ Required project  (6)

▢ Longitudinal experience in the community  (7)

Q50 Does your medical school curriculum offer opportunities to address the following public health competency:  identify quality improvement methods to improve medical care and population health.?

o Yes  (1)

o No  (2)

Display This Question:

If Does your medical school curriculum offer opportunities to address the following public health co... = Yes

Q51 During what year is the content offered? (Choose all that apply.)

▢ Year 1  (1)

▢ Year 2  (2)

▢ Year 3  (3)

▢ Year 4  (4)

Display This Question:

If Does your medical school curriculum offer opportunities to address the following public health co... = Yes

Q52 Please list the course or courses which address this competency.

________________________________________________________________

Display This Question:

If Does your medical school curriculum offer opportunities to address the following public health co... = Yes

Q53 Please indicate the teaching methods used to deliver this content.  (Choose all that apply.)

▢ Didactic Lecture  (1)

▢ Small group discussion focusing on review of the literature  (2)

▢ Small group discussion focusing on a clinical vignette  (3)

▢ Brief experiential learning opportunity in the community  (4)

▢ Structured experience with a local health system  (5)

▢ Required project  (6)

▢ Longitudinal experience in the community  (7)

Q54 My school is interested in expanding the public health curriculum for our students. o Strongly agree  (1)

o Somewhat agree  (2)

o Neither agree nor disagree  (3)

o Somewhat disagree  (4)

o Strongly disagree  (5)

Q55 Completion of this survey was: 

o Extremely easy  (23)

o Somewhat easy  (24)

o Neither easy nor difficult  (25)

o Somewhat difficult  (26)

o Extremely difficult  (27)

Q56 The competencies listed were

o Extremely important  (11)

o Very important  (12)

o Moderately important  (13)

o Slightly important  (14)

o Not at all important  (15)

Q57 The questions regarding which course or courses address a specific competency were 

o Extremely important  (23)

o Very important  (24)

o Moderately important  (25)

o Slightly important  (26)

o Not at all important  (27)

Q58 If you would like to provide further comments, please do so here. 

________________________________________________________________

Q59 Thank you again for taking the time to complete this survey.  If you would like to receive communication about the results of this survey, or be listed as a contributor to this work,  please provide your name and contact information here. 

________________________________________________________________

## References

[1] Institute of Medicine (1999). Committee on Measuring the Health of Persian Gulf Veterans. Committee on Measuring the Health of Persian Gulf Veterans.

[2] Marmot M (2005). Social determinants of health inequalities. Lancet.

[3] Institute of Medicine (2003). Committee on Educating Public Health Professionals for the 21st Century. Who Will Keep the Public Healthy? Educating Public Health Professionals for the 21st Century.

[4] Prescott JE (2011). Exploring the context: contemporary medical education. Am J Prev Med.

[5] Daniel H, Bornstein SS, Kane GC (2018). Addressing social determinants to improve patient care and promote health equity: an American College of Physicians position paper. Ann Internal Med.

[6] Weiss KB, Bagian JP, Wagner R, Nasca TJ (2014). Introducing the CLER pathways to excellence: a new way of viewing clinical learning environments. J Grad Med Educ.

[7] Sales CS, Schlaff AL (2010). Reforming medical education: a review and synthesis of five critiques of medical practice. Soc Sci Med.

[8] Gonzalez CM, Fox AD, Marantz PR (2015). The evolution of an elective in health disparities and advocacy: description of instructional strategies and program evaluation. Acad Med.

[9] (2017). Association of American Medical Colleges. Regional medicine-public health education centers (RMPHEC/RMPHEC-GME). https://www.aamc.org/initiatives/diversity/portfolios/cdc/aamcbased/rmphec/. Published 2017. Accessed.

[10] (2017). AMA creating the medical school of the future. https://www.ama-assn.org/education/creating-medical-school-future. Published 2013. Accessed.

[11] Chappel D, Maudsley G, Bhopal R, Ebrahim S (2008). Public Health Education for Medical Students: A Guide for Medical Schools. Cambridge: Department of Public Health and Primary Care, University of Cambridge.

[12] Tyler IV, Hau M, Buxton JA (2009). Canadian medical students' perceptions of public health education in the undergraduate medical curriculum. Acad Med.

[13] Finkelstein JA, McMahon GT, Peters A, Cadigan R, Biddinger P, Simon SR (2008). Teaching population health as a basic science at Harvard Medical School. Acad Med.

[14] Vela MB, Kim KE, Tang H, Chin MH (2008). Innovative healthcare disparities curriculum for incoming medical students. J Gen Intern Med.

[15] Kerkering KW, Novick LF (2008). An enhancement strategy for integration of population health into medical school education: employing the framework developed by the healthy people curriculum task force. Acad Med.

[16] Chamberlain LJ, Wang NE, Ho ET, Banchoff AW, Braddock CH, Gesundheit N (2008). Integrating collaborative population health projects into a medical student curriculum at stanford. Acad Med.

[17] McNeal MS, Blumenthal DS (2011). Innovative ways of integrating public health into the medical school curriculum. Am J Prev Med.

[18] Associate of American Medical Colleges (2009). Associate of American Medical Colleges. The 2009 state physician workforce data book. The 2009 State Physician Workforce Data Book.

[19] (2018). U.S. Census Bureau. Florida passes New York to become the nation’s third most populous state, census bureau reports. Accessed.

[20] (2015). Liaison Committee on Medical Education. Accredited medical schools in the US. http://lcme.org/directory/accredited-u-s-programs/. Accessed February 9, 2015.

[21] Maeshiro R, Johnson I, Koo D (2010). Medical education for a healthier population: reflections on the Flexner report from a public health perspective. Acad Med.

